# Emerging roles of ATR beyond DNA damage repair: orchestrating transcriptional reprogramming during epithelial-to-mesenchymal transition

**DOI:** 10.1172/JCI207798

**Published:** 2026-08-17

**Authors:** Aida Mestre-Farrera, Zhimin Hu, Jing Yang

**Affiliations:** 1 Department of Pharmacology, Moores Cancer Center, and; 2 Department of Pediatrics, UCSD, La Jolla, California, USA.

## Abstract

The ability of cancer cells to transition between epithelial and mesenchymal states, a process known as epithelial-to-mesenchymal transition (EMT), is a key driver of cancer metastasis and therapy resistance. While ataxia telangiectasia and Rad3-related (ATR) kinase was originally characterized as a responder to DNA damage and replication stress, recent discoveries implicate a critical role for ATR in EMT and metastasis. Two pivotal studies published in this issue of *JCI* provide key insights into how ATR intersects with EMT transcriptional reprogramming. Patel et al. demonstrated that ATR prevented R-loop accumulation at EMT-related gene loci, thereby facilitating the transcriptional reprogramming necessary for EMT as well as tumor growth and metastasis. Tu et al. further uncovered a role for ATR in ECM stiffness–induced EMT, which was associated with an immunosuppressive tumor microenvironment. Together, these studies highlight important therapeutic implications for ATR targeting in the context of metastasis and therapy resistance.

## ATR signaling in cellular homeostasis and cancer progression

Maintaining genomic integrity is vital for cellular homeostasis, particularly in highly proliferative cells subjected to replication stress (1, 2). Ataxia telangiectasia and Rad3-related (ATR), a serine/threonine kinase, serves as a central regulator of this process. Upon activation by ssDNA at stalled replication forks (3, 4), ATR phosphorylates downstream effectors, such as Checkpoint Kinase 1 (CHK1), to induce cell cycle arrest, stabilize replication forks, and facilitate DNA repair (5, 6). The ATR/CHK1 pathway plays a dual role in malignancy: while loss of ATR function drives genomic instability during tumor initiation, advanced tumors often develop an increased dependency on ATR/CHK1 signaling to mitigate replication stress and sustain rapid proliferation (7, 8).

Traditionally recognized as a guardian of genomic stability, ATR is increasingly understood to play roles in cancer that extend beyond canonical DNA damage responses. Emerging evidence suggests it contributes substantially to tumor progression via epithelial-to-mesenchymal transition (EMT). EMT is a highly dynamic and reversible program wherein epithelial cells lose apical-basal polarity and cell-cell adhesion while acquiring mesenchymal traits, including enhanced motility, stem-like properties, and therapy resistance (9). Rather than a binary switch, EMT exists as a spectrum of intermediate or hybrid states that grant tumor cells the plasticity to adapt to microenvironmental stress and facilitate recurrence (10). This program is orchestrated by key EMT transcription factors, such as SNAIL1/2, TWIST1, and ZEB1/2, in response to biochemical cues like TGF-β and physical signals, including mechanical forces from the ECM (11).

Beyond their classical roles in maintaining genome integrity, DNA damage response pathways are now recognized as active modulators of EMT, effectively connecting genome integrity with tumor cell plasticity. In the next section, we examine 2 pivotal studies that expand the role of ATR in regulating EMT. These findings underscore the emerging intersection of DNA damage responses, EMT plasticity, and their collective potential as therapeutic targets in cancer progression.

## ATR-dependent R-loop suppression and transcriptional reprogramming promote EMT

Genomic instability frequently stems from collisions between transcription and DNA replication machineries, leading to the formation of stable RNA:DNA hybrids called R-loops. These structures impede replication fork progression and eventually cause DNA damage (12, 13). Such replication stress is highly relevant to cellular states that demand extensive transcriptional reprogramming.

In this issue of the *JCI*, Patel et al. demonstrated that the extensive transcriptional rewiring during TGF-β–induced EMT generated profound transcription-associated stress and genomic instability (14). This instability was characterized by aberrant R-loop accumulation at key EMT-related loci, such as SNAI1. The authors identified ATR as a critical factor in resolving these R-loop–mediated replication conflicts, thereby enabling the transcriptional reprogramming necessary for EMT. Conversely, ATR blockade in EMT-engaged cells resulted in persistent R-loop accumulation, elevated DNA damage, and repression of SNAI1 and other transition-related genes. These deficits translated to impaired cell migration in vitro and diminished primary and metastatic tumor growth in vivo. Collectively, these findings establish ATR as a vital safeguard of both genomic integrity and transcriptional remodeling, highlighting ATR inhibition as a promising strategy for targeting tumor plasticity.

## ECM stiffness stabilizes ATR protein to promote EMT

Emerging evidence indicates that ATR is activated not only by canonical replication stress and R-loop formation, but also by microenvironmental cues, such as ECM rigidity. In a second study in this issue, Tu and colleagues demonstrated that increased matrix stiffness enhanced ATR protein stability via USP21-mediated deubiquitination, revealing a regulatory mechanism for ATR (15). This stabilized ATR subsequently phosphorylated SUN2, a component of the linker of nucleoskeleton and cytoskeleton complex, which in turn facilitates the nuclear translocation of β-catenin and promotes EMT.

ATR-mediated EMT fostered an immunosuppressive tumor microenvironment, marked by increased recruitment of PMN myeloid-derived suppressor cells and a concomitant reduction in antitumor CD103^+^ dendritic cells. Supporting this, ATR inhibition was shown to attenuate immunosuppression, EMT, and metastasis in immunocompetent breast cancer models. Furthermore, in treatment-resistant early-stage breast cancer patients from the National Cancer Institute 10291 trial (ClinicalTrials.gov NCT04052555), the ATR inhibitor berzosertib significantly increased circulating HLA-DR^+^ antigen-presenting dendritic cells (15). These findings suggest that ATR inhibition enhances antigen presentation and cytotoxic T cell priming. Collectively, these data indicate that the efficacy of ATR-targeted therapies may be optimized by addressing both ATR’s DNA damage–dependent mechanisms and its role in modulating EMT plasticity and immune suppression.

## Implications and future directions

**Figure 1:**
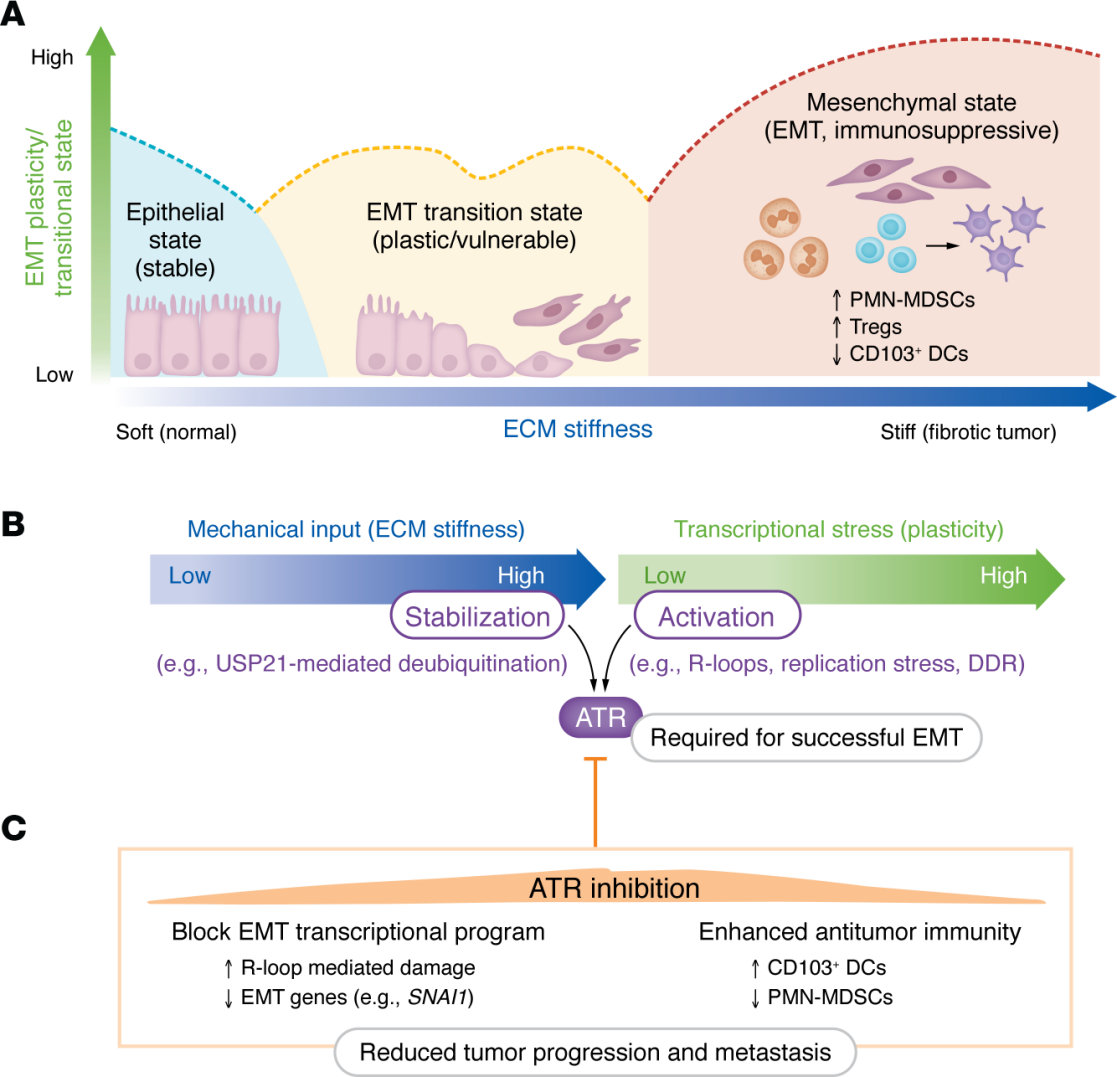
ATR integrates ECM mechanical input and transcriptional stress to regulate EMT plasticity. (**A**) During EMT, epithelial cells lose polarity and acquire traits of mesenchymal cells that support tumor progression and metastasis, including enhanced motility, stem-like properties, and resistance to antitumor therapies. This gradual transition allows tumor cells to adapt to stressors and limitations of the tumor microenvironment. (**B**) Patel et al. (14) showed that during tumor development, tumor cells undergoing EMT experienced transcriptional stress and R-loop accumulation, which activated ATR. In parallel, Tu et al. (15) found that increasing ECM stiffness stabilized ATR via USP21-mediated deubiquitination, which further enhanced EMT transition. At high ECM stiffness, tumor cells transitioned toward a mesenchymal state, gained invasiveness, and promoted an immunosuppressive tumor microenvironment, characterized by increased PMN myeloid-derived suppressor cells (PMN-MDSCs) and Tregs and decreased CD103^+^ dendritic cells (DCs). DDR, DNA damage response. (**C**) Pharmacological inhibition of ATR in the work of Patel et al. and Tu et al. disrupted EMT-associated transcriptional programs and enhanced antitumor immunity, respectively. Collectively, these effects suppressed tumor progression and metastasis.

Collectively, the studies by Patel et al. and Tu et al. propose a comprehensive model for ATR in tumor progression and metastasis, positioning it as a central hub for EMT induction triggered by convergent biochemical and mechanical stimuli from the microenvironment. The model proposes that during tumor progression, activated cancer-associated fibroblasts (CAFs) secrete TGF-β, initiating EMT-related transcriptional reprogramming. This rapid transcription reprogramming activity generates R-loop–associated DNA damage that necessitates ATR-mediated repair. Simultaneously, CAFs modify the ECM to increase stiffness, which cooperates with TGF-β to activate transcription factors like TWIST1 and SNAI1 to further boost EMT transcriptional reprogramming. High ECM stiffness further stabilizes ATR protein, driving β-catenin localization and resolving R-loop–induced damage. These reinforcing positive feedback loops demonstrate how mechanical and biochemical cues converge on ATR to orchestrate the EMT program (Figure 1).

These groundbreaking studies prompt critical questions regarding the noncanonical roles of ATR, which extend beyond its established function in DNA damage repair. During the transcriptional reprogramming associated with EMT, it remains to be elucidated how ATR selectively localizes to TGF-β–induced EMT gene promoters instead of other transcriptionally active genes. Although SUN2 is established as a key ATR substrate for β-catenin nuclear translocation, the established role of the LINC complex in modulating heterochromatin in response to mechanical cues suggests a more extensive function beyond β-catenin. This indicates that ATR may orchestrate a broader mechanosensitive EMT program in response to ECM rigidity, extending its influence well beyond canonical DNA damage repair pathways.

The EMT program contributes substantially to tumor metastasis and therapeutic resistance across various human malignancies. Emerging evidence further underscores the critical role of EMT in driving tumor-mediated immune suppression, particularly within the context of ATR inhibition. While several ATR inhibitors are currently undergoing clinical evaluation in combination with chemotherapy and radiotherapy (16, 17), the discovery of ATR as a central hub in EMT regulation suggests a broader therapeutic window. Investigating the impact of ATR inhibitors on diverse forms of therapy resistance may expand their clinical utility beyond canonical DNA damage repair mechanisms.

## Conflict of interest

The authors have declared that no conflict of interest exists.

## Funding support

This work is the result of NIH funding, in whole or in part, and is subject to the NIH Public Access Policy. Through acceptance of this federal funding, the NIH has been given a right to make the work publicly available in PubMed Central.

- National Cancer Institute grants R01CA174869, RO1CA262794, and R01CA268179 (to JY).
- St. Baldrick’s Foundation research grant (to JY).
- Krueger v. Wyeth research award (to JY).
- Tobacco-Related Disease Research Program postdoctoral award T32FT4922 (to AMF).
- Pfizer Oncology–Cell Signaling San Diego postdoctoral fellowship (to ZH).

## Version 1 08/17/2026

Electronic publication
